# Optimal Screening Strategies for Healthcare Associated Infections in a Multi-Institutional Setting

**DOI:** 10.1371/journal.pcbi.1003407

**Published:** 2014-01-02

**Authors:** Aaron C. Miller, Linnea A. Polgreen, Philip M. Polgreen

**Affiliations:** 1 Department of Pharmacy Practice & Science, University of Iowa College of Pharmacy, Iowa City, Iowa, United States of America; 2 Division of Infectious Diseases, Department of Internal Medicine, University of Iowa Carver College of Medicine, Iowa City, Iowa, United States of America; Pennsylvania State University, United States of America

## Abstract

Health institutions may choose to screen newly admitted patients for the presence of disease in order to reduce disease prevalence within the institution. Screening is costly, and institutions must judiciously choose which patients they wish to screen based on the dynamics of disease transmission. Since potentially infected patients move between different health institutions, the screening and treatment decisions of one institution will affect the optimal decisions of others; an institution might choose to “free-ride” off the screening and treatment decisions of neighboring institutions. We develop a theoretical model of the strategic decision problem facing a health care institution choosing to screen newly admitted patients. The model incorporates an SIS compartmental model of disease transmission into a game theoretic model of strategic decision-making. Using this setup, we are able to analyze how optimal screening is influenced by disease parameters, such as the efficacy of treatment, the disease recovery rate and the movement of patients. We find that the optimal screening level is lower for diseases that have more effective treatments. Our model also allows us to analyze how the optimal screening level varies with the number of decision makers involved in the screening process. We show that when institutions are more autonomous in selecting whom to screen, they will choose to screen at a lower rate than when screening decisions are more centralized. Results also suggest that centralized screening decisions have a greater impact on disease prevalence when the availability or efficacy of treatment is low. Our model provides insight into the factors one should consider when choosing whether to set a mandated screening policy. We find that screening mandates set at a centralized level (i.e. state or national) will have a greater impact on the control of infectious disease.

## Author Summary

Healthcare associated infections are a major cause of morbidity and mortality. Screening patients on admission to the hospital may reduce prevalence by identifying infected individuals; infected individuals can then be treated or isolated to prevent further spread. Because screening is costly, institutions must weigh the benefits of reduced prevalence against the costs of screening. However, patients move between institutions carrying disease with them; consequently, when choosing who to screen, institutions must also consider the rates at which neighboring institutions screen patients as well. We develop a theoretical model that describes this strategic decision process. Using this model we are able to analyze the screening decision problem along three dimensions: (1) how disease specific parameters, such as the effectiveness of treatment, influence the optimal screening level, (2) how the degree of centralization in screening policy (e.g. local, state or federal) influences the optimal screening level, and (3) how these two sets of factors combine to influence the optimal screening level. Our model highlights factors to consider when choosing to implement screening policy, and results are of use to policy makers wishing to reduce the prevalence of infectious disease.

## Introduction

Hospital associated infections represent a major cause of morbidity and mortality [1]. Disease screening can facilitate efforts to reduce disease prevalence through the treatment of infected patients or reducing transmission within a healthcare setting. Because screening and treatment are expensive, institutions must choose a level of screening to balance the associated costs with the benefits of lower disease prevalence. However, in a setting where patients flow back and forth between different institutions, the prevalence in one institution will directly affect others that it shares patients with. This implies that the screening decisions of one institution can impact the prevalence and, consequently, the optimal screening levels of other institutions with a shared patient population. One can imagine a situation in which a single institution could “free-ride” off of a low prevalence rate induced by neighboring institutions that rigorously screen and treat patients.

In such a strategic environment, it is important to determine wether or not individual health institutions will choose a screening level that is socially optimal. The economic literature is full of examples where strategic settings induce suboptimal decision making; in some cases, only a social planner can induce the socially optimal decision. In the case of screening, this might imply setting a screening policy at a national, state or regional level. Indeed, a handful of countries outside of the US have already implemented nationally mandated screening policies for diseases such as MRSA [2] [3], and policy makers and health professionals in the US have also called for universal or mandatory screening of MRSA [4] [5]. Such screening mandates arise because policy makers believe that individuals or healthcare organizations may not make optimal screening decisions on their own.

The goal of this paper is to provide insight into the strategic disease-screening process along with the factors that should be considered when choosing an optimal screening policy. To do so, we develop a theoretical model of disease screening and analyze behavior across a wide range of disease and screening parameters. Properly studying this strategic screening problem requires a game theoretic model that can incorporate the underlying dynamics of disease transmission. In order to capture both disease dynamics and strategic decision making, we draw on an existing body of literature that has sought to combine epidemiologic compartmental models of disease transmission with economic models of decision making [6]–[12]. Specifically, this study develops a theoretical model by incorporating a Susceptible-Infected-Susceptible (SIS) compartmental model into a game-theoretic setting where institutions must choose the number of patients to screen, given the screening decisions of other institutions that they share patients with. Using this framework, we can analyze how incentives are influenced by changing the number of decision makers, i.e. making institutions more or less autonomous in setting their own screening policy. In the concept of a game, changing the level of autonomy is effectively equivalent to changing the number of players in the game while the underlying population remains fixed. Our model extends previous literature, which has analyzed how the number of decision makers influences optimal decision making in disease settings [9], by allowing for (1) a variable number of decision makers in a fixed patient population, (2) movement of patients between health care institutions and (3) variability of treatment efficacy. Thus, we develop a general model intended to be broadly applicable to the disease-screening process.

## Methods

We begin by considering an epidemiological model of disease flow with subpopulations that consist of patients in health institutions and a non-institutional population. In each of these subpopulations there will exist a number of infected individuals that transmit disease to unaffected individuals. As individuals move back and forth between the different subpopulations, e.g. patients going to the hospital, they carry with them disease and may subsequently infect susceptible populations. We next consider how institutions might control this spread of disease, namely with screening, and develop a game theoretic model to represent this problem. Institutions can screen patients on admission in order to reduce transmission or treat those infected. Since screening and treatment are costly, institutions must consider a tradeoff when choosing an optimal screening level. However, each of these costs will depend on the level of disease present, and as a result, the optimal choice will depend on the decision of other institutions. Finally, we develop a framework to analyze how changing the level of unit autonomy can be analyzed in this setting. This model largely extends the work of Smith et al. (2005) by analyzing screening decisions in a fixed population with a variable number of decision makers.

### Epidemiological Disease Model

To model the spread of disease within a population composed of different healthcare institutions and a non-institutional community, we build an SIS compartmental model of disease. In this model, individuals within each subpopulation are assumed to exist in one of two states: (1) *susceptible*, the disease is not present, and (2) *infected*, the disease is present. Susceptible individuals may receive the disease and become infected, whereas infected individuals may transmit the disease or recover and return to the susceptible population. We can represent the proportion of a subpopulation *k* that is infected by 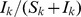, where 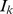 and 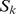 are the number of infected and susceptible individuals, respectively. We are interested in two types of subpopulations, the various healthcare institutions that screen patients and the community. We assume there are *N* different institutions and a single community. Let 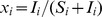 and 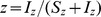 represent the disease prevalence in a particular health institution *i* and the community, respectively.

There are two ways in which screening may influence disease prevalence, namely treatment and transmission. Let 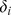 be the percentage of the patient population that institution *i* decides to screen. If treatment for a disease exists, screening may identify infected individuals that can be treated and returned to the susceptible state. We denote the daily treatment recovery rate within an institution as a function of its screening level by 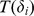. This function will depend on the efficacy of treatment and screening. The only restrictions we place on its functional form are that it is assumed to be nondecreasing in the screening level, 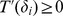, and must not be greater than the rate of screening, 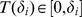. This first assumption implies that greater screening does not lead to less recovery from treatment, and the second implies that patients are screened before they are treated. We must also account for the fact that individuals may recover from the disease naturally without treatment. Let *λ* denote the natural recovery rate of the disease. These two parameters imply that the recovery from the disease in a health institution *i* is given by 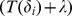.

In addition to treatment, screening may also facilitate reduced transmissibility. For example, cases that are identified may be isolated or additional hygienic measures may be taken in areas around such cases. Let 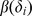 denote the institutional transmission rate as a function of the screening rate. The change in disease prevalence at health institution *i* from individuals becoming infected via transmission is given by 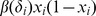. We assume the transmission rate is a non-increasing function of the unit's screening level, 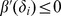. In addition, transmission may occur in the community as well. Let 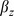 denote the transmission rate in the community. Both the transmission and treatment functions are developed in further detail and given specific functional forms below.

**Figure 1:**
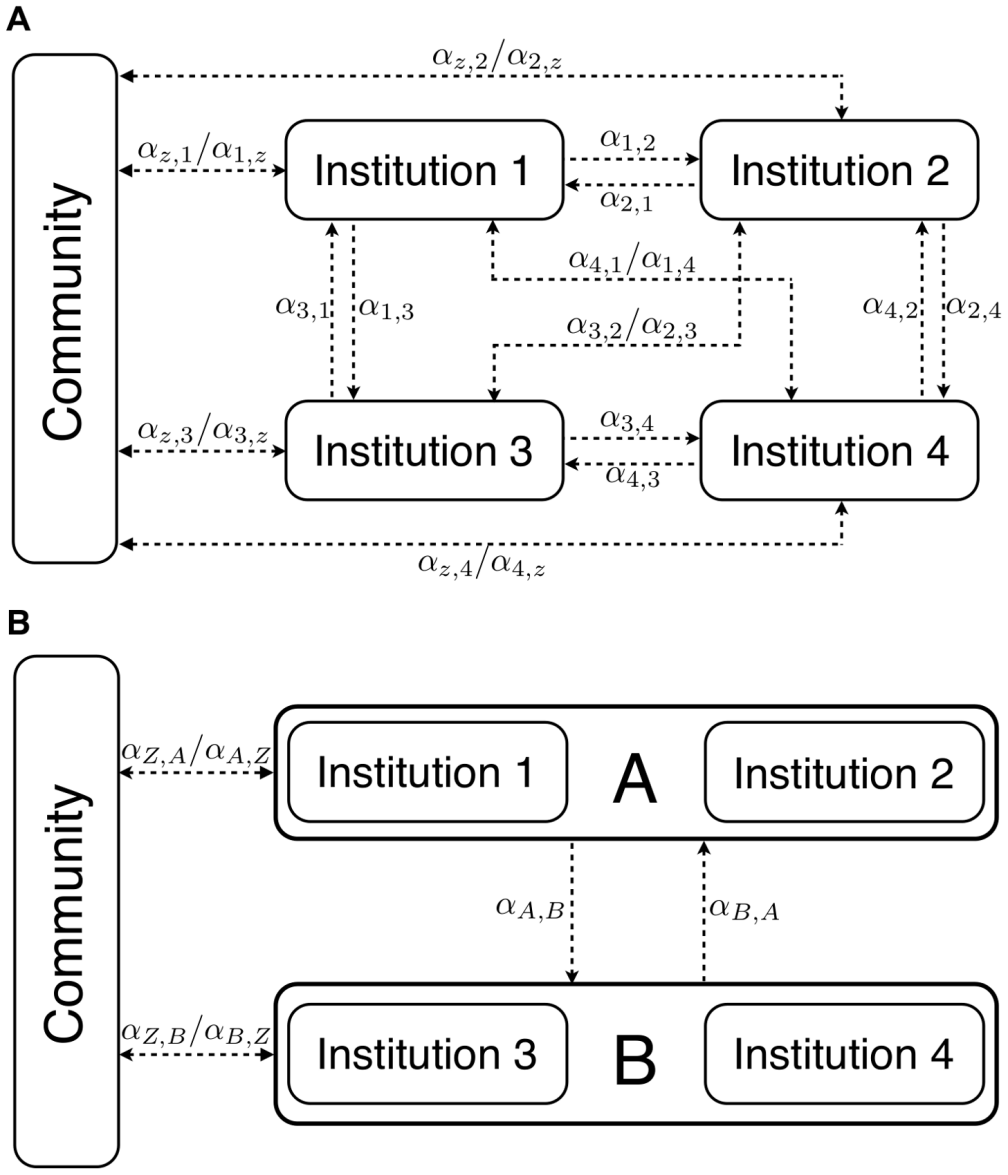
Model of patient flow. In Figure A, the direction of patient movement between various health institutions and the community, along with the associated flow parameters, are depicted in a setting with four institutions. In Figure B, these institutions have been clustered into two DUs, and the flow parameters between the DUs and the community are shown. Comparing Figure A to Figure B illustrates how patient movement between institutions becomes internalized into the flow parameters depicted in equation 6 when institutions are clustered into a focal DU and second DU.

Finally, disease prevalence within each institution as well as the community will be affected by the movement of infected individuals among the various populations. This movement is described by two types of flow parameters: (1) the rate at which individuals move out of a particular subpopulation, and (2) the direction of movement to the various subpopulations. First, let 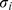 denote the rate of patient turnover at institution *i* (i.e. 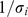 is the average patient length of stay in institution *i*); we assume this rate is constant among the different institutions. Similarly, let 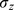 denote the turnover rate in the community (i.e. rate of admission to health institutions). Second, let 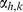 denote the proportion of patients transferred from subpopulation *h* to subpopulation *k*, where *h* and *k* include both health institutions and the community (the order of subscripts reflects the direction of patient movement). Figure 1A provides a summary of this patient movement in a setting with four different institutions. Since institutions are assumed to be homogenous, the proportion of movement between the various health institutions must be equivalent, i.e. 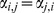 where *i* and *j* are different institutions, and 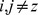. Similarly, the homogeneity assumption implies that the proportion of movement between each institution and the community must be identical, i.e. 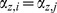 for all 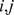. In equilibrium these flow parameters must capture all patient movement, i.e. 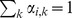 where *k* includes all other institutions and the community. Therefore, these flow rates will be fixed by the number of institutions 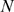 and the proportion of movement from the community 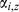; flow parameters can then be rewritten as 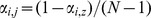.

Given the parameters described above along with each institution's chosen screening level, the dynamics of disease prevalence within each institution, 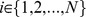, and the community can be represented by the following system of differential equations:
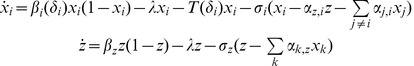
(1)where 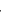 and 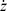 represent the derivatives of 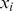 and *z* with respect to time. We can solve this system to obtain disease prevalence in a single institution *i* as a function of *i*'s screening level, the screening level of all other institutions and time. Thus, disease prevalence in institution *i* can be expressed by the function 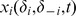, where 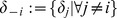 represents the set of screening policies set by all other institutions. It can be shown that this function is decreasing in *i*'s screening level and the screening level of all other institutions.

### Economic Model and Game

The economic decision problem facing each institution is to choose the level of screening that minimizes the net present value of all future costs. Institutions must tradeoff the costs associated with screening versus the cost associated with increased disease prevalence. Screening costs in institution *i*, at a single point in time, are given by the following:
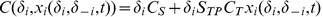
(2)where 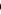 is the cost of screening per patient screened, 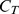 is the per patient cost of treatment, and 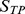 is the screening true positive rate. Although this functional form specifically implies that only cases that correctly test positive receive treatment, the values for the parameters 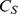 and 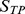 or 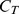 can be adjusted to capture the possibility that false positives also receive treatment. Moreover, the value for treatment costs 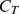 can also be designed to include costs associated with secondary or confirmatory screening (see [13]).

Although screening is costly, there are also costs associated with disease prevalence. For example, increased prevalence within an institution may lead to greater transmission, make treating unrelated diseases more costly, require additional hospital personnel and, if conditions become severe enough, diminish the institution's reputation. Let the costs associated with disease prevalence be given by γ, so the total costs associated with prevalence in institution *i* are given by 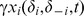.

Given these two cost sources, an institution must choose a single level of screening that minimizes its net present value of all future costs. Because disease dynamics and the associated prevalence evolve over time, as described by system (1), total costs are derived by integrating over changes in disease prevalence across time. Thus, the institution's decision problem can be stated as the following:
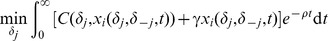
(3)where *ρ* is the economic discount rate. We can see from this equation that an institution's optimal screening level will be dependent on the screening levels set in all other institutions, 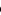. This implies that choosing an optimal screening level is a game; an institution's optimal screening level will depend on the screening strategy taken by its opponents (i.e. the other institutions). We can define an institution's best response function, given the screening level set by its opponents, by the following:
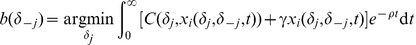
(4)Because all institutions are homogeneous, we are primarily interested in finding solutions that constitute a symmetric pure strategy Nash equilibrium, i.e. a fixed screening level that is mutually optimal when all institutions respond identically. We obtain the symmetric equilibrium by finding a fixed point of this best response function, i.e. a value 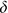 such that 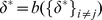.

### Modeling Institutional Autonomy

The purpose of this study is not to determine a specific institution's optimal screening level given a set of parameters, but rather to describe what happens to the optimal level as institutions are made more or less autonomous in setting their own screening policy. We can simulate the effects of varying the level of autonomy by clustering sets of individual institutions into groups where screening policy in each group is then set by a single decision maker. We will refer to these groups of institutions as Decision Units (DUs) and assume there are a total of 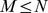 DUs covering all institutions. If screening policy were to be set at a national level we would have 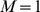 DU, whereas if screening were set at a state level we would have 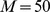 DUs. Thus, when units are perfectly autonomous 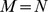. Notice that we can find the socially optimal screening level, the single screening level that minimizes total societal costs across all institutions, by setting 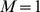. For simplicity, we assume that these DUs are homogenous and each contains the same number of institutions, i.e. the number of institutions in each DU is 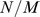. We also assume that a single decision maker managing each DU will consider the costs over all the institutions within the DU when choosing a screening level. Thus, the problem facing a decision maker managing a set of institutions 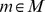 is simply to choose a screening level that minimizes (3) summed across all institutions contained in *m*.

Because we are only looking for symmetric pure strategy equilibria, the above problem can be reduced to considering only two DUs, namely a focal DU and a second DU composed of all other DUs. Since all DUs are homogenous, solving the problem from the perspective of the focal unit must then solve the problem for all units. We begin by reducing system (1) to that of a focal unit *A*, a cluster of all other DUs *B* and a community *z*. This system governing disease dynamics then becomes the following:
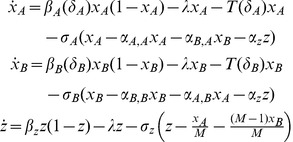
(5)Where 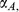 and 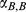 denote transfers made between institutions within the same DU, 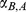 and 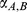 denote transfers between institutions in different DUs, and 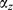 denotes the proportion of transfers to a single institution that are from the community. This system is stated in terms of only two DUs in order to solve for the symmetric equilibrium, but the flow parameters (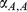, 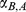, etc.) account for the fact that patients flow between institutions both within the same DU and among DUs. Figure 1B depicts an example of such patient flow in a setting with four institutions and two DUs. Comparing the flow parameters between Figures 1A and 1B demonstrates how the patient flow between individual institutions becomes internalized when patient movement is modeled in terms of a focal DU and all other DUs. In equilibrium these flow parameters between DUs must satisfy the following:
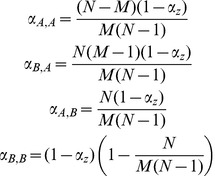
(6)Because all institutions and DUs are assumed to be homogenous, the only flow parameter that must be specified in this system is the proportion of admitted patients coming from the community.

Given the above system of disease dynamics, we can solve for disease prevalence in the focal DU as a function of its own screening level 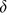, the screening level of its opponent 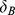, the total number of DUs *M* and time *t*, i.e. 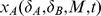. This allows us to rewrite the best response function from (4) for the focal DU by the following:
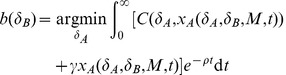
(7)Finally, we obtain the equilibrium of interest by finding a fixed point of this best response function, i.e. a value 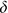 such that 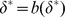 where 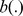 is given by (7). Note: from this point forward we will refer to *M* as a measure of institutional autonomy, where the level of institutional autonomy can be thought to be increasing with the number of DUs given by *M*.

This SIS compartmental model lacks a closed form solution; thus, we solve the model numerically while exploring a realistic space of parameter values. Table 1 contains a summary of the model parameters along with the range of values that was explored. To solve the model numerically, it is first necessary to specify functional forms for both the transmission and the treatment functions. We use the following form to estimate the transmission function:
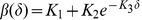
(8)where 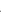, 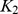, and 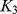 are all positive, constant, parameter values that can be calibrated using disease transmissibility data. This functional form has a number of useful properties. First, it exhibits diminishing marginal returns from screening. We assume that increasing the screening level when the initial screening level is very low has a greater impact on transmissibility than when the initial level is very high. For example, initial screening may identify areas of the hospital or specific patient populations to focus on. This also allows us to capture the fact that patients may be screened on their probability of infection. Second, this function can be calibrated to converge to a minimum level of transmissibility with perfect screening, given by the value 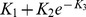. We assume that even when all patients are screened and treated, there may still exist a positive probability of transmission to susceptible patients. For example, individuals may contract disease from contact with surfaces in a hospital setting. Finally, the parameters 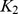 and 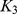 can be used to calibrate this function to the rate at which transmission declines with increased screening and treatment. Because our objective here was to provide a general understanding of screening decision process applicable to a wide range of diseases, we explored a range of values for 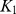, 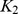, and 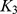 and analyzed the effects of varying each of these values individually and simultaneously. This analysis is described in further detail in the sensitivity analysis section below. In addition, we analyzed the impact of using a linear transmission function in place of (8); a description of this analysis can be found in Appendix S1.

**Table 1:** Model parameters and range of values explored.

Parameter	Description	Range Explored
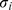	Patient turnover rate at institution *i*	1/10–1
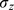	Hospital admission rate in the community	1/1000–1/10
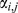	Proportion of patients transferred from institution *i* to institution *j*	Set by 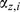
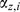	Proportion of patients transferred from outside population to institution *i*	.1–.9
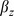	Transmission rate in outside population	0–.5
*λ*	Natural recovery rate without treatment	1/1000–1/10
*τ*	Treatment efficacy rate	0–1
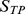	Sensitivity of screening test	0–1
γ	Prevalence cost of disease	1–7
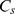	Cost of screening	.1–2.5
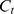	Cost of treatment	.1–2.9
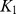	Limiting value of transmission function (as 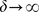 )	.0001–.3
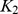	Maximal value of transmission function (for 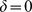 )	.1–.9
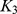	Slope coefficient for transmission function	1.5–8
*ρ*	Economic discount rate	.01–.15
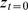	Initial disease prevalence (in outside population)	.0001–.3

Finally, we use the following function to model the treatment recovery rate:
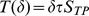
where *τ* is the treatment efficacy rate and, as before, 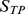 is the true positive rate of screening. With this function we assume that anyone who is identified as having the disease gets treated and recovers at a rate proportional to the efficacy of treatment.

## Results

**Figure 2:**
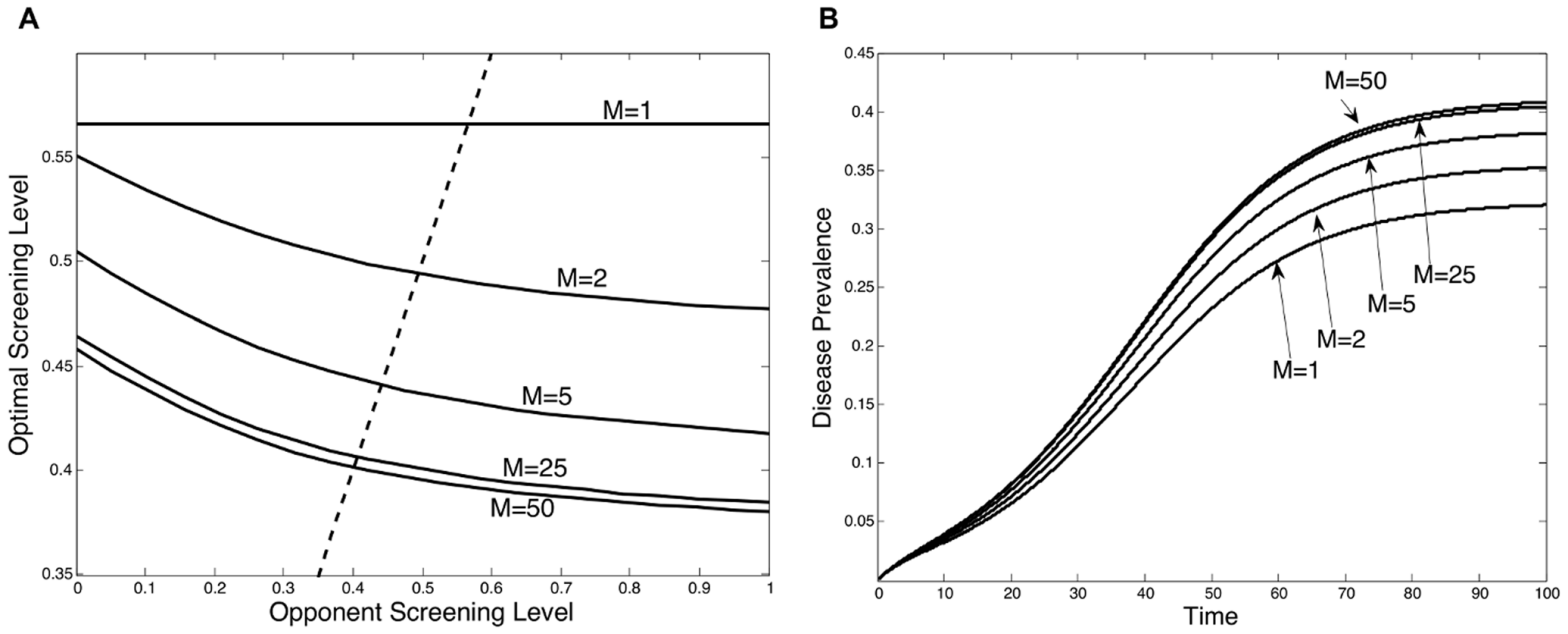
Changing the screening autonomy level. The strategic screening levels and associated disease prevalence are influenced by the degree of institutional autonomy *M*. (A) Best response curves depict an individual institution's optimal screening rate *δ* as a function of the screening level 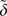 chosen by the other institutions (i.e. its opponents). The equilibrium screening level, occurring at each curve's intersection with the dashed line, increases as autonomy *M* decreases. (B) Disease prevalence over time, corresponding to the equilibrium screening levels in Figure A, is greater with increased autonomy *M*.

We analyzed our model across a wide space of different parameter values meant to capture a wide variety of diseases and screening settings. Throughout our analysis we consistently obtained two main results. First, increasing the level of screening autonomy (*M*) shifts the best response curve downward, which results in a lower equilibrium screening level. The change in optimal screening can be seen from Figure 2A. In this figure the symmetric equilibrium, which occurs where each best response curve intersects the dashed fixed-point line, can be seen to be monotonically decreasing as the level of autonomy increases. As a result, this decline in equilibrium screening will cause disease prevalence to increase; this can be seen from Figure 2B. The disease prevalence over time depicted in Figure 2B corresponds to the equilibrium screening levels shown in Figure 2A. These results indicate that as the level of institutional autonomy increases, the equilibrium screening level may decrease, and disease prevalence may increase. However, the effect of changing the level of autonomy is not uniform; Figures 2A and B both show that decreasing the autonomy level from five to two has a much greater impact than moving from 50 to 25. In other words, as the level of autonomy increases, the marginal effect from adding more decision makers becomes much smaller. This same result holds across the range of parameter values. These findings are consistent with previous research that has found that spending on hospital infection control decreased with the size of the hospital population [9].

**Figure 3:**
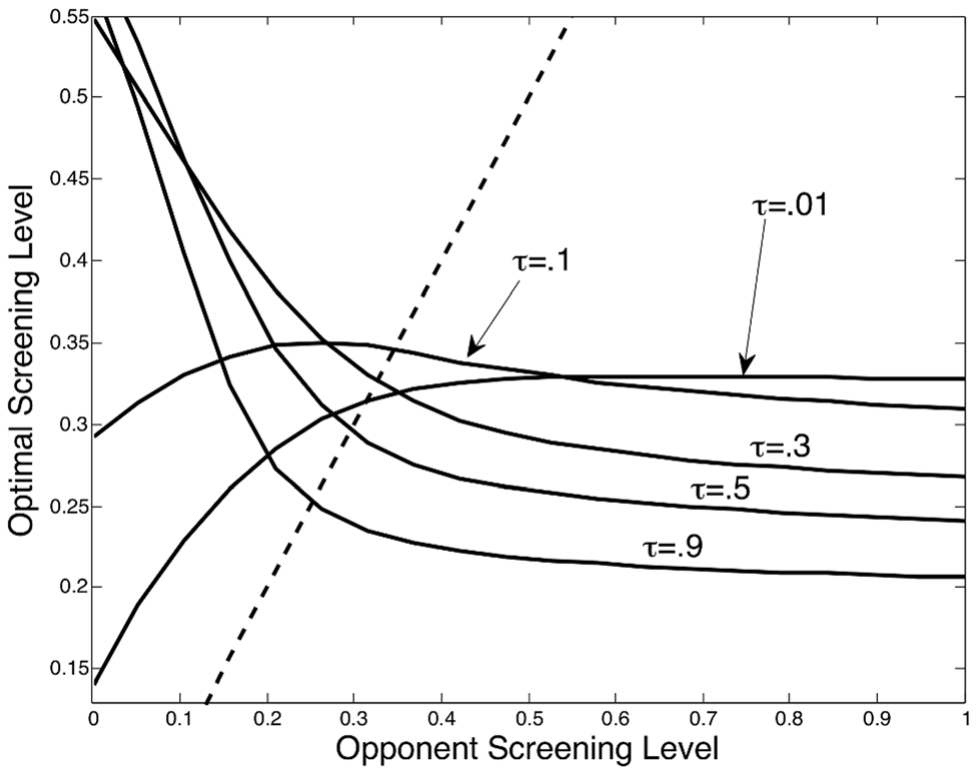
Changing the level of treatment efficacy. The shape of the best response curve and the equilibrium screening level are influenced by the level of treatment efficacy *τ*. As treatment becomes more effective (*τ* increases), the best response curve shifts from an increasing concave function to a decreasing convex function. The equilibrium screening level decreases as treatment becomes more effective from 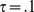 to 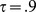.

Our second finding is that the availability and efficacy of treatment (*τ*) has a major influence on the shape of the best response curve. When treatment is less effective, the downward slope of the best response curve diminishes and, in some cases, this curve may even become upward sloping. Figure 3 depicts how the shape of the best response curve changes with treatment efficacy. When treatment is less effective, or not available at all, an institution's best response curve may resemble an increasing function of its opponent's screening level; this is depicted in Figure 3 where 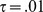. In this situation preventing the spread of disease through transmission becomes the primary means of reducing prevalence. Reducing transmission is more effective when institutions cooperate and all engage in screening. Thus, as more institutions engage in screening, screening becomes relatively more effective, which provides an incentive for other institutions to engage in screening as well. On the other hand, when treatment is more effective, and becomes the primary means of reducing prevalence, cooperation in screening between institutions is no longer necessary to reduce prevalence. In this latter situation, when a majority of institutions engage in screening, the prevalence of disease may be effectively reduced to the point where a single institution may find it beneficial to “free-ride” off the screening decisions of its neighbors by simply choosing to screen less. Thus, as treatment becomes more effective the best response curve generally becomes downward sloping; this can be seen in Figure 3 for 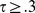.

**Figure 4:**
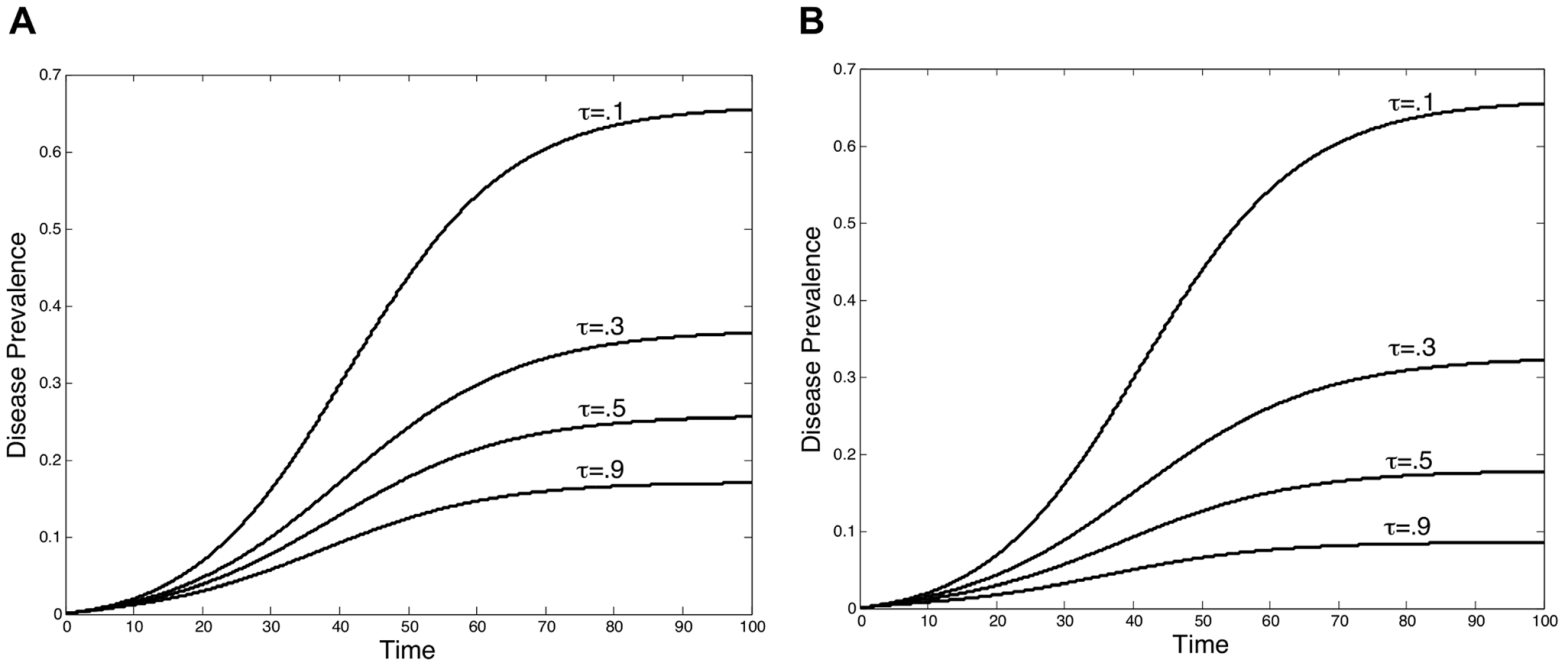
Disease prevalence with different levels of treatment efficacy. When treatment efficacy increases, some of the decrease in disease prevalence is lost due to a decline in screening. Figure A depicts the prevalence corresponding to the equilibrium levels of screening provided in Figure 3. While prevalence decreases with the increase in treatment efficacy, it does not decline by as much as it would have if screening did not decline as well. In Figure B, disease prevalence is shown for each level of treatment efficacy if institutions were to screen at the higher screening level associated with the equilibrium for 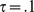, depicted in Figure 3.

This changing shape of the best response curve induced by the level of treatment efficacy implies that, for some parameter values, as treatment becomes more effective the equilibrium screening level may actually decrease. An example of this can be seen in Figure 3 where the equilibrium screening level systematically decreases as treatment efficacy increases from 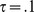 to 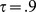. Although the optimal screening level may decline with an increase in treatment efficacy, the disease prevalence in the population is still likely to decrease. In Figure 4A, disease prevalence is depicted across time for the different equilibrium screening levels shown in Figure 3. This figure demonstrates that an increase in treatment efficacy allows a lower prevalence level to be attained even when the screening level is reduced. However, some of this added benefit from the increased treatment efficacy gets crowded out by the drop in screening. Figure 4B depicts the disease prevalence that would have occurred if institutions continued screening at the higher equilibrium screening level, corresponding to when 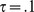, when treatment became more effective.

**Figure 5:**
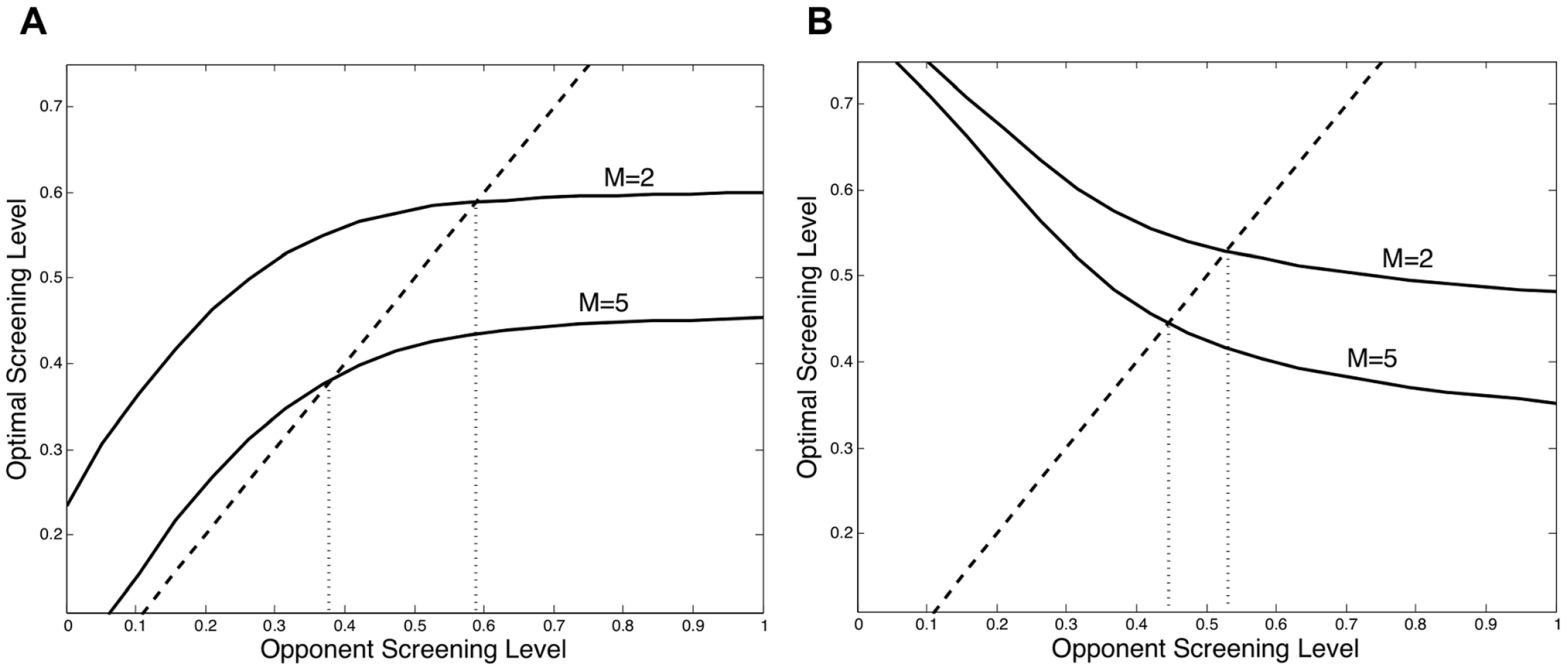
Crowding out with increased treatment efficacy. Treatment efficacy and the associated shape of the best response curve determine how much a change in the autonomy level will influence the equilibrium screening level. In Figure A, treatment is less effective (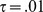) and a decrease in screening autonomy increases the equilibrium screening level by much more than in Figure B where treatment is more effective (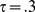).

If we combine the changing shape of the best response curve with the fact that increased autonomy leads to a downward shift in the best response curve, we find that increasing screening autonomy may have a much greater impact on screening, and consequently prevalence, when treatment is less effective. This result occurs because, when treatment is less effective, the fixed point line may intersect the best response curve at a point where the best response curve is increasing. When treatment is more effective, the fixed point line intersects the best response curve at a decreasing point on the curve. This result can be seen in Figure 5. In Figure 5A, when treatment is less effective, an increase in the level of autonomy from two to five causes the screening rate to decline from 58% to around 37%, whereas when treatment is more effective, as in Figure 5B, the screening rate only declines from around 54% to around 45%.

**Figure 6:**
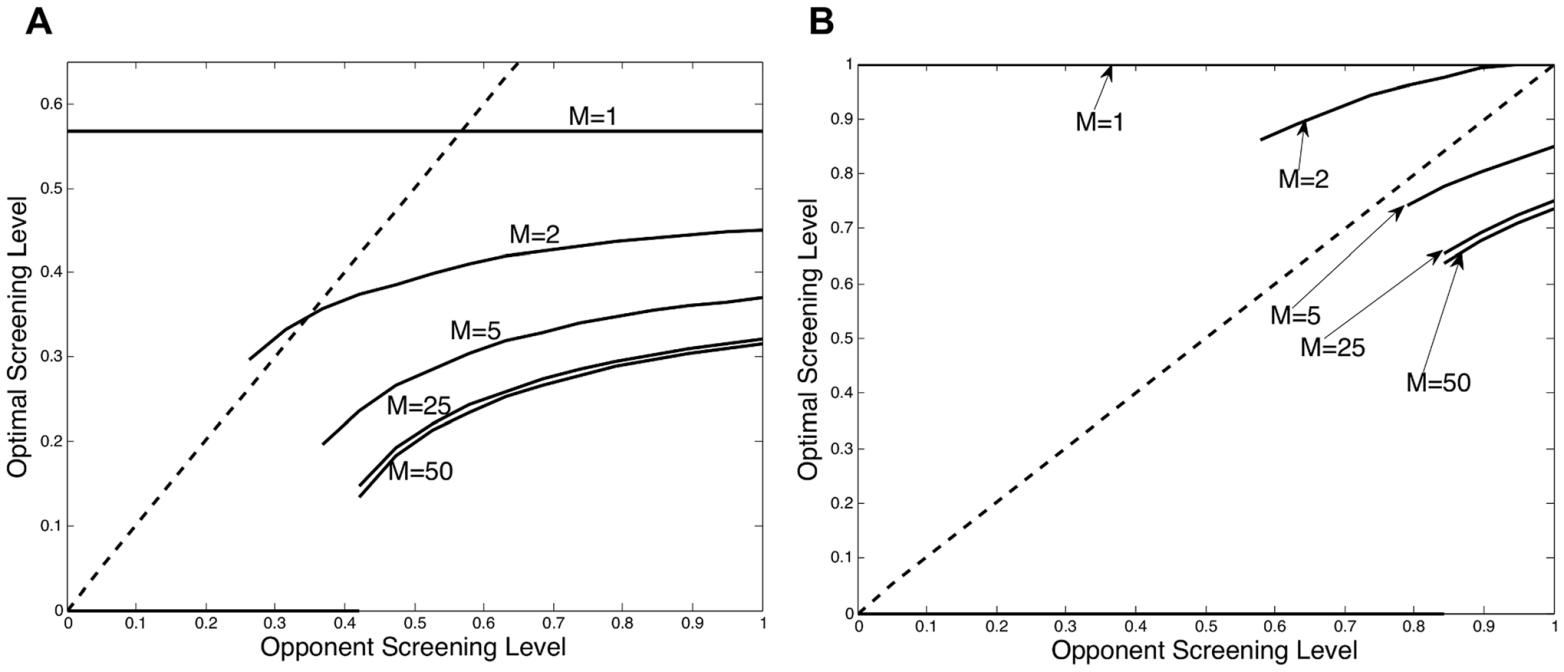
No screening equilibria. Under some disease parameter values, when treatment is less effective and the best response curve is upward sloping, increasing the degree of screening autonomy may lead a “no-screening” equilibrium, where institutions simply choose not to screening for disease. In both figures, screening takes place at a positive value when the level of screening autonomy is low (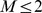) but no screening occurs as autonomy increases (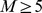). In Figure A, decreasing autonomy from five to two DUs results in 35% of patients being screening instead of none, while in Figure B, the same decrease in autonomy results in all patients being screened.

One final result that we obtain is that for some parameter values when treatment is less effective, increasing the level of autonomy may lead to a “no-screening” equilibrium. Figure 6 depicts two cases where a greater autonomy level actually shifts the best response curve away from the fixed point line leading to a no-screening equilibrium. In both cases, when 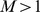 a screening rate of 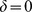 becomes a possible equilibrium point because the point (0,0) is always a fixed point on the best response curve. In Figure 6A, when there are one or two DUs, institutions screen around 55 or 35% of patients, respectively, but as autonomy increases, institutions screen zero patients. Figure 6B depicts an even more extreme case where institutions screen all patients, 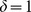, when there are one or two DUs but screen no patients as autonomy increases. These results demonstrate that when treatment is less effective screening may only be cost effective if all institutions are screening at a high enough rate, and this may only occur with less institutional autonomy when screening levels are coordinated across institutions.

### Sensitivity Analysis

To ensure that the results presented above were not tied to a specific set of parameter values we performed a sensitivity analysis by exploring a wide range of potential parameter values. Because we wanted our results to be generalizable to both a variety of possible diseases and a range of screening settings, we chose to explore a wide range of values for each parameter rather than fit the parameters to specific empirical estimates. This parameter space was intended to cover all empirically plausible values a parameter could represent. In Table 1, each of the specific parameters in our model is described along with the range of values that were explored. For each parameter, a univariate analysis was performed by varying the parameter over this space. Additionally, a number of multivariate sensitivity analyses were performed on sets of related variables by simultaneously varying multiple parameters over this space. A detailed description of this sensitivity analysis can be found in Appendix S1. Throughout this sensitivity analysis we tested the strength of our major findings by comparing the changing shape of the best response curve as we varied the level of treatment efficacy *τ* and number of DUs *M*.

The results of our sensitivity analysis confirmed that our two major findings hold across the entire parameter space explored. First, Figures S1, S2, S3, S4, S5, S6, S7, S8, S9, S10 show that along the entire parameter space, a decrease in treatment efficacy was associated with a transformation of the best response curve in such a way that its downward trend diminished. For most of the parameter values explored, this decrease in treatment efficacy resulted in a transition of the best response curve from downward sloping to upward sloping. However, while the downward trend of the best response curve did diminish in all cases we explored, for some parameter values the decline in treatment efficacy is not enough to cause the curve to become upward sloping (see Figures S5 or S10). Second, Figures S11, S12, S13, S14, S15, S16, S17, S18, S19, S20 depict the effect of changing the number of DUs across the parameter space and for different levels of treatment efficacy. These figures show that across the entire range of parameters analyzed, an increase in the number of decision makers was associated with a decline in the equilibrium screening level. This sensitivity analysis demonstrates that while the disease dynamics of the underlying compartmental model may be affected by the specific parameter values, the basic outcomes of the screening game appear to be stable across a wide range of parameter values. This finding suggests that our results are applicable to a wide range of diseases and settings.

## Discussion

This study provides insights into the optimal level of disease screening in a strategic multi-institutional setting where potentially infected patients move back and forth between different screening institutions. This model allows us to analyze how the optimal level of screening is influenced by the number of decision makers, i.e. the level of institutional autonomy in choosing a screening level, as well as various disease parameters. The overarching message of this study is that having more decision makers in the screening process leads to less screening and increased disease prevalence. This means that mandated screening levels, either at a state or national level, may be more effective at controlling the spread of disease than simply allowing individual institutions to set their own screening level. Having a greater number of decision makers increases the opportunity to free-ride, and, in a setting where infected patients spread disease while moving between institutions, the actions of a single decision maker will be less effective than those of a coordinated group. In such multi-institutional settings, coordination of actions, which can only occur under a limited number of decision makers, essentially makes screening cheaper and more effective.

This basic result, that more decision makers may lead to worse outcomes, is fairly intuitive and is consistent with previous research [8]. In fact, an understanding of this result has likely been the motivation for efforts to implement screening mandates for certain diseases. However, this study adds two significant refinements to the current literature, which have additional policy implications. First, the marginal benefit that is provided by decreasing the number of decision makers declines as the number of decision makers increases. Moving from 50 to 25 decision makers was shown to have a much smaller relative benefit than moving from five decision makers to two. Consequently, efforts that only slightly reduce autonomy (e.g. setting policy across affiliated institutions) may have little discernible impact on screening and disease prevalence. This fact might encourage policy makers to adopt a “go big or go home” type of strategy, by only choosing to implement screening mandates that move to a single or very limited number of decision makers.

The second major refinement that this study provides is to highlight the fact that the added benefit of moving to a smaller number of decision makers is largely dependent on the availability and efficacy of treatment. The marginal benefit from reducing the number of decision makers is much greater for diseases with no, or less effective, treatment. In some cases, if a disease is less treatable and institutions are given too much autonomy, they may end up choosing not to screen at all. This occurs because with diseases that are less treatable, strategies aimed at reducing transmission become the primary method for reducing prevalence, and the effectiveness of such strategies is largely dependent on the level of coordination between institutions. If treatment is not an option, a single institution will have a much harder time trying to prevent transmission when neighboring institutions are not doing the same and will, consequently, give up. Therefore, mandated screening policy becomes much more important for diseases that are less treatable. For diseases such as Klebsiella pneumonia with New Delhi metal-beta-lactamase-1 (NDM-1) [14] or Acinetobacter baumannii [15], it may be much more important for screening policy to be mandated by a central planner. Thus, the emergence of multi-drug resistant organisms where there are no, or very few, antibiotics available for treatment leads to a situation where a central screening policy is more effective.

One limitation of this study that deserves mention is the assumption of a homogeneous setting. In our model, institutions and DUs were assumed to be identical, and patients were assumed to be homogeneously mixed in order to guarantee the existence of a pure strategy equilibrium. In reality, institutions are not perfectly homogeneous, and patient transfers often vary greatly between institutions. This is especially true when comparing different types of wards within a hospital or between different types of institutions. In such a heterogeneous setting, it is likely that different institutions will have different screening incentives and, consequently, choose different optimal screening levels. For example, a large hospital with a high patient flow may find it preferable to screen at a greater rate than a smaller hospital. However, amending the model to include a heterogeneous institutional population would very likely eliminate the existence of pure strategy equilibria for many parameter values. A heterogeneous screening model is also likely to lead to the existence of multiple mixed strategy equilibria. Because our goal in this paper was to provide a general understanding of how the level of institutional autonomy can influence the screening decision process and because such mixed strategy equilibria are often difficult to interpret intuitively, we chose to model institutions homogeneously. Therefore, the results presented here should be thought of as providing an intuition for how the number of decision makers and the level of treatment efficacy impact screening incentives in general, rather than providing an empirical estimate of how specific institutions will behave.

Although screening results have been presented in terms of economic optimality, it is worth noting that such optimality specifically refers to the theoretical optimization problem present in our model; in reality, the empirically optimal screening level may be dependent on factors that our model did not account for. One example where this distinction may be important is when interpreting the result that an increased number of decision makers may lead to less socially optimal screening outcomes. Our model did not take into account the costs associated with establishing and enforcing mandated screening levels. Mandating screening across institutions at a national or even state level is likely to entail a number of political, administrative and enforcement costs that were not considered in this model. Policy makers will need to consider such costs along with the implications presented here when choosing the best policy. However, one result from this study that may shed light on this policy making process is the fact that the marginal benefit of implementing a mandatory screening policy will be greater when it involves a smaller number of individual decision makers. This result directly implies that if the costs of imposing a screening mandate are roughly the same for establishing either a local or federal mandate, it will be a much more cost effective decision to establish a national rather than local mandate. Moreover, the costs of establishing a local mandate will need to be significantly lower than those associated with a national mandate in order for the local mandate to be a preferable option.

## Supporting Information

Appendix S1 **Detailed description of sensitivity analysis.**
(PDF)

Figure S1**Sensitivity analysis of *τ* across parameter space for **
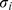
**.** Across the range of values explored for 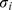, a lower level of treatment efficacy *τ* was associated with either a diminished downward slope or a shift to an upward sloping best response curve. Best response curves are shown for different values of 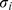, when 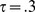 (left) and 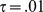 (right).(TIF)

Figure S2**Sensitivity analysis of *τ* across parameter space for **
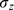
**.** Across the range of values explored for 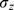, a lower level of treatment efficacy *τ* was associated with either a diminished downward slope or a shift to an upward sloping best response curve. Best response curves are shown for different values of 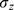, when 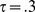 (left) and 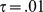 (right).(TIF)

Figure S3**Sensitivity analysis of *τ* across parameter space for **
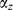
**.** Across the range of values explored for 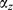, a lower level of treatment efficacy *τ* was associated with either a diminished downward slope or a shift to an upward sloping best response curve. Best response curves are shown for different values of 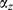, when 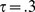 (left) and 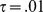 (right).(TIF)

Figure S4**Sensitivity analysis of *τ* across parameter space for **
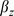
**.** Across the range of values explored for 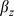, a lower level of treatment efficacy *τ* was associated with either a diminished downward slope or a shift to an upward sloping best response curve. Best response curves are shown for different values of 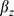, when 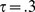 (left) and 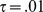 (right).(TIF)

Figure S5**Sensitivity analysis of *τ* across parameter space for *λ*.** Across the range of values explored for *λ*, a lower level of treatment efficacy *τ* was associated with either a diminished downward slope or a shift to an upward sloping best response curve. Best response curves are shown for different values of *λ*, when 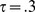 (left) and 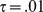 (right).(TIF)

Figure S6**Sensitivity analysis of *τ* across parameter space for **
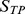
**.** Across the range of values explored for 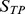, a lower level of treatment efficacy *τ* was associated with either a diminished downward slope or a shift to an upward sloping best response curve. Best response curves are shown for different values of 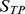, when 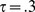 (left) and 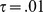 (right).(TIF)

Figure S7**Sensitivity analysis of *τ* across parameter space for cost parameters.** Across the range of values explored for the cost parameters γ, 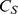, and 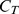, a lower level of treatment efficacy *τ* was associated with either a diminished downward slope or a shift to an upward sloping best response curve. For each of the cost parameters, best response curves are shown for a range of parameter values, when 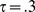 (left) and 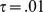 (right). These figures also demonstrate that units of cost are arbitrary and the best response curve is affected by the ratio of prevalence costs, *γ*, to screening and treatment costs, 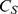 and 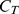.(TIF)

Figure S8**Sensitivity analysis of *τ* across parameter space for the parameters of the transmission function.** Across the range of values explored for the parameters of the transmission function, 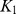, 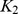, and 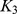, a lower level of treatment efficacy *τ* was associated with either a diminished downward slope or a shift to an upward sloping best response curve. For each of the parameters of the transmission function, best response curves are shown for a range of parameter values, when 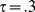 (left) and 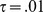 (right).(TIF)

Figure S9**Sensitivity analysis of *τ* across parameter space for *ρ*.** Across the range of values explored for *ρ*, a lower level of treatment efficacy *τ* was associated with either a diminished downward slope or a shift to an upward sloping best response curve. Best response curves are shown for different values of *ρ*, when 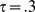 (left) and 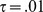 (right).(TIF)

Figure S10**Sensitivity analysis of *τ* across parameter space for **
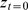
**.** Across the range of values explored for 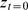, a lower level of treatment efficacy *τ* was associated with either a diminished downward slope or a shift to an upward sloping best response curve. Best response curves are shown for different values of 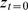, when 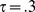 (left) and 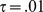 (right).(TIF)

Figure S11**Sensitivity analysis of *M* across parameter space for **
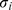
**.** Across the range of values explored for 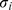, a lower level of screening autonomy *M* was associated with an upward shift of the best response curve and a higher equilibrium screening level. Best response curves are shown for different values of 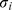 when 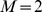 (solid line) and 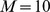 (dashed line) at both 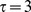 (left) and 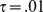 (right).(TIF)

Figure S12**Sensitivity analysis of *M* across parameter space for **
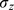
**.** Across the range of values explored for 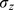, a lower level of screening autonomy *M* was associated with an upward shift of the best response curve and a higher equilibrium screening level. Best response curves are shown for different values of 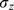 when 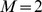 (solid line) and 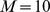 (dashed line) at both 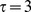 (left) and 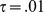 (right).(TIF)

Figure S13**Sensitivity analysis of *M* across parameter space for **
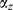
**.** Across the range of values explored for 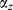, a lower level of screening autonomy *M* was associated with an upward shift of the best response curve and a higher equilibrium screening level. Best response curves are shown for different values of 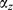 when 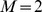 (solid line) and 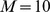 (dashed line) at both 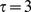 (left) and 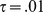 (right).(TIF)

Figure S14**Sensitivity analysis of *M* across parameter space for **
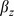
**.** Across the range of values explored for 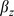, a lower level of screening autonomy *M* was associated with an upward shift of the best response curve and a higher equilibrium screening level. Best response curves are shown for different values of 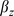 when 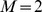 (solid line) and 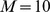 (dashed line) at both 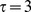 (left) and 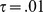 (right).(TIF)

Figure S15**Sensitivity analysis of *M* across parameter space for *λ*.** Across the range of values explored for *λ*, a lower level of screening autonomy *M* was associated with an upward shift of the best response curve and a higher equilibrium screening level. Best response curves are shown for different values of *λ* when 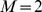 (solid line) and 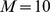 (dashed line) at both 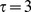 (left) and 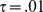 (right).(TIF)

Figure S16**Sensitivity analysis of *M* across parameter space for **
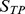
**.** Across the range of values explored for 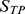, a lower level of screening autonomy *M* was associated with an upward shift of the best response curve and a higher equilibrium screening level. Best response curves are shown for different values of 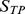 when 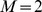 (solid line) and 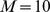 (dashed line) at both 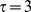 (left) and 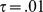 (right).(TIF)

Figure S17**Sensitivity analysis of *M* across parameter space for cost parameters.** Across the range of values explored for the cost parameters γ, 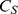, and 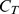, a lower level of screening autonomy *M* was associated with an upward shift of the best response curve and a higher equilibrium screening level. For each of these cost parameters, best response curves are shown for different values of *ρ* when 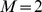 (solid line) and 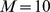 (dashed line) at both 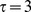 (left) and 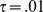 (right). These figures continue to demonstrate that units of cost are arbitrary and the best response curve is affected by the ratio of prevalence costs, γ, to screening and treatment costs, 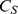 and 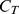.(TIF)

Figure S18**Sensitivity analysis of *M* across parameter space for parameters of the transmission function.** Across the range of values explored for the parameters of the transmission function, 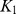, 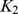, and 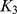, a lower level of screening autonomy *M* was associated with an upward shift of the best response curve and a higher equilibrium screening level. For parameters of the transmission function, best response curves are shown for different values of *ρ* when 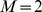 (solid line) and 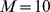 (dashed line) at both 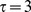 (left) and 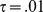 (right).(TIF)

Figure S19**Sensitivity analysis of *M* across parameter space for *ρ*.** Across the range of values explored for *ρ*, a lower level of screening autonomy *M* was associated with an upward shift of the best response curve and a higher equilibrium screening level. Best response curves are shown for different values of *ρ* when 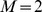 (solid line) and 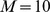 (dashed line) at both 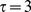 (left) and 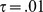 (right).(TIF)

Figure S20**Sensitivity analysis of *M* across parameter space for **
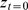
**.** Across the range of values explored for 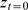, a lower level of screening autonomy *M* was associated with an upward shift of the best response curve and a higher equilibrium screening level. Best response curves are shown for different values of 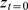 when 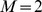 (solid line) and 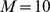 (dashed line) at both 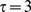 (left) and 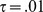 (right).(TIF)

Figure S21**Multivariate sensitivity analysis across parameter space for *λ* and **
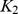
**.** Values for the two parameters primarily associated with affecting the disease reproductive ratio, namely *λ* and 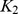, were varied simultaneously. Our two primary findings were consistent across the values explored in this multivariate analysis: (1) a lower level of screening autonomy *M* was associated with an upward shift of the best response curve, and (2) a lower level of treatment efficacy *τ* was associated with a diminished downward slope of the best response curve.(TIF)

Figure S22**Multivariate sensitivity analysis across transmission function parameters, for **
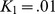
**.** Values for the transmission function parameters 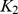 and 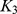 were varied simultaneously while 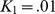. Our two primary findings were consistent across the values explored in this multivariate analysis: (1) a lower level of screening autonomy *M* was associated with an upward shift of the best response curve, and (2) a lower level of treatment efficacy *τ* was associated with a diminished downward slope of the best response curve.(TIF)

Figure S23**Multivariate sensitivity analysis across transmission function parameters, for **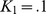. Values for the transmission function parameters 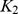 and 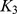 were varied simultaneously while 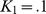. Our two primary findings were consistent across the values explored in this multivariate analysis: (1) a lower level of screening autonomy *M* was associated with an upward shift of the best response curve, and (2) a lower level of treatment efficacy *τ* was associated with a diminished downward slope of the best response curve.(TIF)

Figure S24**Effect of a linear transmission function.** Best response curves are shown across a range of values for 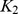 with the nonlinear transmission function (left) and a linear transmission function (right). The transition to a linear transmission function results in a reduction of the curvature and an upward shift of the best response curves. However, our main findings were consistent even when the linear transmission function was used.(TIF)

Table S1 **Description of model parameters and corresponding baseline values used for sensitivity analysis.**
(PDF)
