# Supplementary material for: Optimal Screening Strategies for Healthcare Associated Infections in a Multi-Institutional Setting
Source: PLoS Comput Biol. 2014 Jan 2;10(1):e1003407. doi: 10.1371/journal.pcbi.1003407 (PMC3879151; doi:10.1371/journal.pcbi.1003407)
Supplement: Appendix S1 — Detailed description of sensitivity analysis. (PDF) [file pcbi.1003407.s001.pdf]

APPENDIX S1

1. Sensitivity Analysis

The goal of this sensitivity analysis was to explore the widest possible range of parameter values to ensure that the results presented in our manuscript were applicable to a variety of diseases and screening settings. We varied parameters individually, or in limited subsets. These baseline values are provided in Table S1. With the exception of the patient turnover parameters σ_z and σ_i, none of the other parameters were specifically chosen to reflect empirical disease specifications. The baselines for these patient turnover parameters were obtained using the 2009 Healthcare Cost and Utilization Project (HCUP) Nationwide Inpatient Sample, where patients had a mean length of stay of around 4.6 days, and the 2005-2011 HCUP State Inpatient Databases for California, where patients had approximately 296 days between inpatient visits. These turnover parameters were set to empirical values for two reasons. First, our model specifically applies to an inpatient population. Second, these turnover parameters are multiplied by patient flow parameters (e.g. α_z) in the model and, thus, their specific effect on the best response curve can largely be captured by exploring such flow parameters.

For the remaining parameters, which can be described as disease or screening specific, baseline values were not set to empirical specifications but rather to explore the widest plausible parameter space. We used a baseline selection process intended to observe the largest amount of variation that could be attributed to each parameter, while attempting to prevent any one baseline parameter value from "masking" the effects of another. To do so we analyzed how each parameter affected the equilibrium screening level over the entire parameter space, and then chose a baseline value for each parameter that approximately produced the "median" effect on the screening equilibrium, across different levels of τ. This general selection process took place in the following steps. First, we determined a range for each parameter (see Table 1) that we believed to cover all possible values a parameter could assume. Then for each parameter we initially set a baseline value at the middle of this range. Next we analyzed the range of best response curves that were produced as we varied each parameter between the bounds of this parameter space. Finally, we adjusted the baseline value, if necessary, to the value that appeared to produce an equilibrium at the approximate middle of the range in response curves that were generated. We did this while also taking into consideration the impact of different levels of treatment efficacy. While this baseline selection process required a certain level of subjectivity in selecting the baseline parameter values that represented the median equilibrium effect, we are confident our choice

of baseline values allowed us to explore a wide enough parameter space to fully characterize the outcomes of interest; the results described in our manuscript were consistent across the entire range of parameter values explored.

1.1. **Univariate Sensitivity Analysis.** We first conducted a univariate sensitivity analysis by systematically varying each parameter in our model. For each parameter, we analyzed best response curves and equilibrium screening levels as we varied the parameter along the space described in Table 1, while the remaining parameters were held fixed to their baseline values. We compared these effects between different levels of treatment efficacy τ and different numbers of DUs M. Supporting Figures S1-S10 show the effects of varying treatment efficacy between $\tau = .3$ and $\tau = .1$. In Figure S7 the various cost variables (γ, C_S, and C_T) have been combined to show that the units of cost are arbitrary; the optimization problem depends on costs of prevalence γ relative to the costs of screening C_S and treatment C_T. Similarly, in Figure S8 the graphs depicting the best response curves corresponding to variation of the parameters of the transmission function (K_1, K_2, and K_3) have been combined because these parameters jointly determine the shape of the transmission function. Supporting Figures S11-S20 show the effects of varying the number of decision makers from $M = 2$ to $M = 10$. As before the graphs associated with costs and the parameters of the transmission function have been combined in Figures S17 and S18, respectively.

As described in our manuscript, our primary findings were consistently supported throughout the entire parameter space we explored. Decreasing the treatment efficacy rate was associated with a transformation of the best response curve; lower efficacy rates were associated with either a diminished downward trend or a transition to an upward sloping best response curve. Likewise, increasing the number of decision makers was associated with a drop in the best response curve and a lower equilibrium screening level. We also analyzed different values for the number of DUs and confirmed that the marginal effect of increasing the number of DUs diminished as more were added.

1.2. **Multivariate Sensitivity Analysis.** In addition to the univariate sensitivity analysis described above, we also conducted two multivariate sensitivity analyses on sets of related variables. First, because the parameters K_1, K_2, and K_3 jointly determine how transmission is affected by the screening level, we thought it valuable to explore the effect of varying these parameters simultaneously. Figures S22 and S23 show the results of varying K_2 and K_3 when $K_1 = .01$ and $K_1 = .1$, respectively. Second, because the reproductive ratio in the underlying compartmental model is largely determined by the values of λ and K_2 we also thought it would be valuable to analyze the effect of varying these two parameters in tandem. Figure S21 shows the effects of varying both λ and K_2 simultaneously. These two multivariate sensitivity analyses extend the findings of the univariate analysis and

demonstrate that the primary findings of our study hold across the entire range of parameter values we analyzed.

2. Linear Transmission Function

In order to determine the robustness of our results in relation to the functional form of the transmission function $\beta(\delta)$, we also analyzed the effect of specifying a linear transmission function in place of Equation (8). In order to make an accurate comparison between the linear and non-linear transmission function, we specified the linear form to have the same transmission rates for the bounds of the screening range $\delta \in [0, 1]$. Therefore, the linear screening function was defined as the following:

$$\beta(\delta) = (K_1 + K_2)(1 - \delta) + (K_1 + K_2 e^{-K_3})\delta$$

Figure S24, demonstrates the effect of specifying the transmission function as a linear function of δ. By comparing the graph on the left (nonlinear) to that on the right (linear) two general effects can be seen. First, the linear specification causes the curvature of the best response curves to be reduced. Second, the linear specification causes the best response curves to shift slightly upward. In general, these two results held across the range of values we explored. Despite the effect that the linear specification had on the best response curve, the major findings of our study were not changed by the choice of a nonlinear transmission function. Changing the number of decision makers or the level of treatment efficacy had the same effect on the best response curve when the transmission function was linearly defined.
