# Supplementary material for: Optimal Screening Strategies for Healthcare Associated Infections in a Multi-Institutional Setting
Source: PLoS Comput Biol. 2014 Jan 2;10(1):e1003407. doi: 10.1371/journal.pcbi.1003407 (PMC3879151; doi:10.1371/journal.pcbi.1003407)
Supplement: Table S1 — Description of model parameters and corresponding baseline values used for sensitivity analysis. (PDF) [file pcbi.1003407.s026.pdf]

Table S1. Baseline Parameter Values

Parameter	Description	Baseline Value
σ_i	Patient turnover rate at institution i	1/4.6
σ_z	Hospital admission rate in the community	1/296
$\alpha_{i,j}$	Proportion of patients transferred from institution i to institution j	set by $\alpha_{z,i}$
$\alpha_{z,i}$	Proportion of patients transferred from outside population to institution i	.5
β_z	Transmission rate in outside population	.1
λ	Natural recovery rate without treatment	1/100
τ	Treatment efficacy (recovery rate following treatment)	varried
S_{TP}	Sensitivity of screening test	.9
γ	Prevalence cost of disease	3
C_s	Cost of screening	.5
C_t	Cost of treatment	1.5
K_1	Limiting value of transmission function (as $\delta \to \infty$)	.01
K_2	Maximal value of transmission function (for $\delta = 0$)	.5
K_3	Slope coefficient for transmission function	5
ρ	Economic discount rate	.05
$z_{t=0}$	Initial disease prevalence (in outside population)	.05
